# Synthesis and Biological Screening of 4-Benzyl-2*H*-phthalazine Derivatives

**DOI:** 10.3390/ph4081158

**Published:** 2011-08-17

**Authors:** Ashraf H.F. Abd El-Wahab, Hany M. Mohamed, Ahmed M. El-Agrody, Mohammed A. El-Nassag, Ahmed H. Bedair

**Affiliations:** 1 Chemistry Department, Faculty of Science, Al-Azhar University, 11884, Nasr City, Cairo, Egypt; 2 Chemistry Department, Faculty of Science, Jazan University, 2097, Jazan, Saudi Arabia; 3 Chemistry Department, Faculty of Medicine, Jazan University, 82621, Jazan, Saudi Arabia; 4 Chemistry Department, Faculty of Science, King Khalid University, 9004, Abha, Saudi Arabia

**Keywords:** phthalazine derivatives, anthracene derivatives, synthesis, antimicrobial activity

## Abstract

Preparation of 4-benzyl-2-substituted phthalazin-1-one derivatives **2-8** is reported. Condensation of 4-benzyl-1-chlorophthalazine (**9**) with a series of different nucleophiles gave 4-benzylphthalazin-1-ylamino derivatives (**10-13** and **16**) and 4-amino-2-[*N*′-(4-benzylphthalazin-1-yl)-hydrazino]-6-arylpyrimidine-5-carbonitriles (**14a**,**b**). Interaction of **9** with ambident anions was also studied. 5-Benzyl-6,6a,12-triazobenzo[*a*]-anthracen-7-one (**15**) is obtained from **9** and anthranilic acid derivatives. Treatment of **16** with (EtO)_3_CH/Ac_2_O under reflux afforded the corresponding ethoxymethylene derivative **17**, while aqueous ammonium hydroxide treatment afforded carboxamide derivative **18**. The structures of the newly synthesized derivatives were confirmed by their elemental analysis, IR, ^1^H NMR, ^13^C NMR and mass spectral studies. Antimicrobial activities of some selected compounds were also studied and some of these were found to exhibit promising effects against Gram-positive and Gram-negative bacteria and fungi.

## 1. Introduction

**Figure 1:**
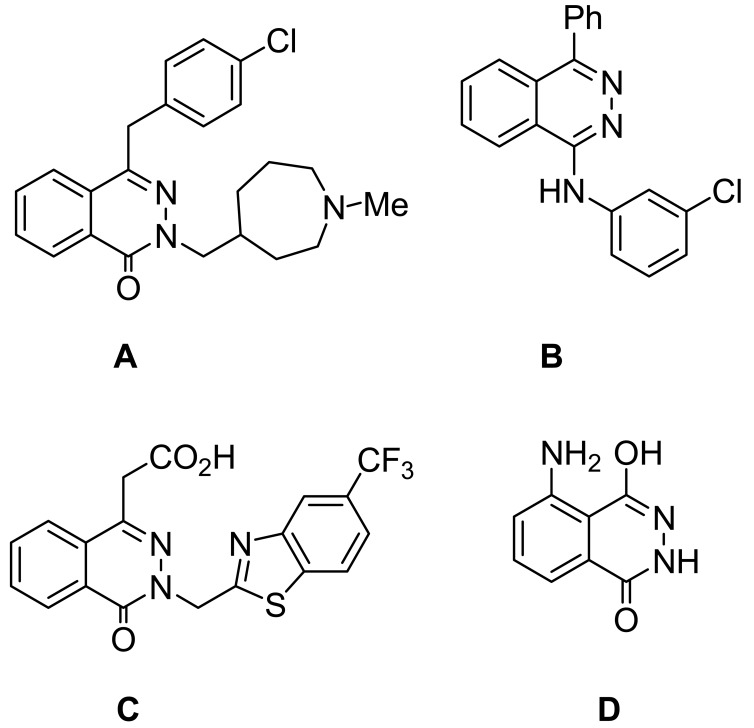
Phthalazine derivatives (azelastin, MY 5445, zopolrestat and luminol).

The phthalazine derivative azelastine (**A**, Figure 1) is an antihistamine used in the treatment of allergic rhinitis [1]. Newer agents are more selective inhibitors of the cGMP-inhibited phosphor diesterase (PDE) and casn be exemplified by phthalazine derivatives like MY5445 (**B**, Figure 1) [2-5]. Zopolrestat (**C**, Figure 1) is a phthalazinone derivative that has been in clinical trials; it inhibits aldose reductase and has potential use in the prevention of retinopathy, neuropathy, and cataract formation in diabetes [6]. The chemiluminescence reactions of luminol (**D**, Figure 1) and related phthalazines have found analytical applications, particularly in biological systems where the inherent signal strength and low signal noise ratio contribute to sensitivity. The hydrogen peroxide/luminol system has been used for the on-line determination by chemiluminescence of nitric oxide in isolated organ perfusates [7-10].

Phthalazine derivatives have been widely applied as therapeutic agents due to their anticonvulsant, cardiotonic, vasorelaxant and anti-inflammatory properties [11-17] in addition to having antimicrobial activity [18].

## 2. Results and Discussion

**Scheme 1:**
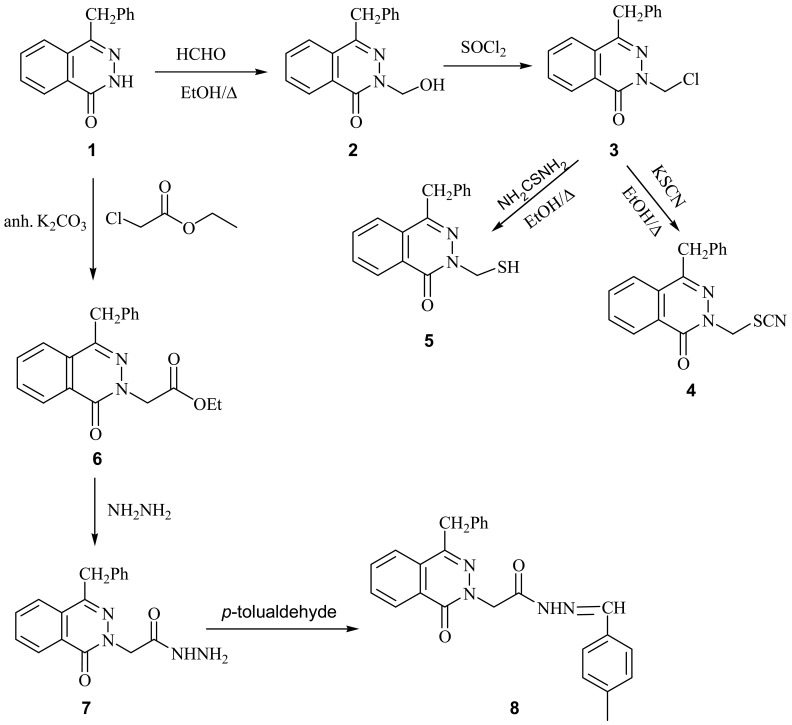
Synthesis of phthalazine-1-one derivatives.

4-Benzyl-2*H*-phthalazin-1-one (**1**) [19] was used as a starting material for this investigation. Reaction of compound **1** with formalin in ethanol afforded 4-benzyl-2-hydroxymethyl-2*H*-phthalazin-1-one (**2**). Treatment of **2** with thionyl chloride afforded the corresponding 4-benzyl-2-chloromethyl-2*H*-phthalazin-1-one (**3**). Interaction of **3** with alcoholic potassium thiocyanate yield the corresponding 4-benzyl-2-thiocyanatomethyl-2*H*-phthalazin-1-one (**4**), while with ethanolic thiourea it afforded 4-benzyl-2-mercaptomethyl-2*H*-phthalazin-1-one (**5**). Treatment of **1** with ethyl chloroacetate in the presence of anhydrous K_2_CO_3_ afforded the corresponding ethyl (2-(4-benzyl-1-oxo-1*H*-phthalazin-2-yl)acetate (**6**). Reaction of **6** with hydrazine hydrate gave the corresponding (4-benzyl-1-oxo-1*H*-phthalazin-2-yl)acetic acid hydrazide (**7**) which was reacted with *p*-tolualdehyde to give the corresponding (4-benzyl-1-oxo-1*H*-phthalazin-2-yl) acetic acid (4-methylbenzylidene) hydrazide (**8**) (Scheme 1).

**Scheme 2:**
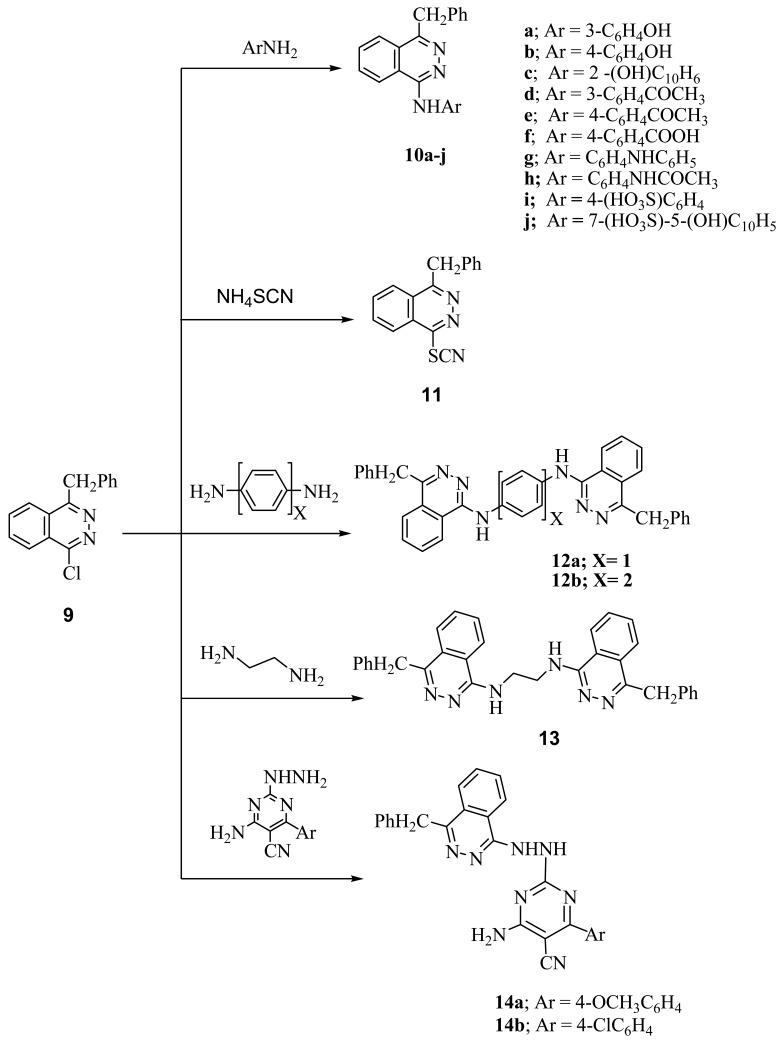
Reactions of 4-benzyl-1-chlorophthalazine with amines derivatives.

**Scheme 3:**
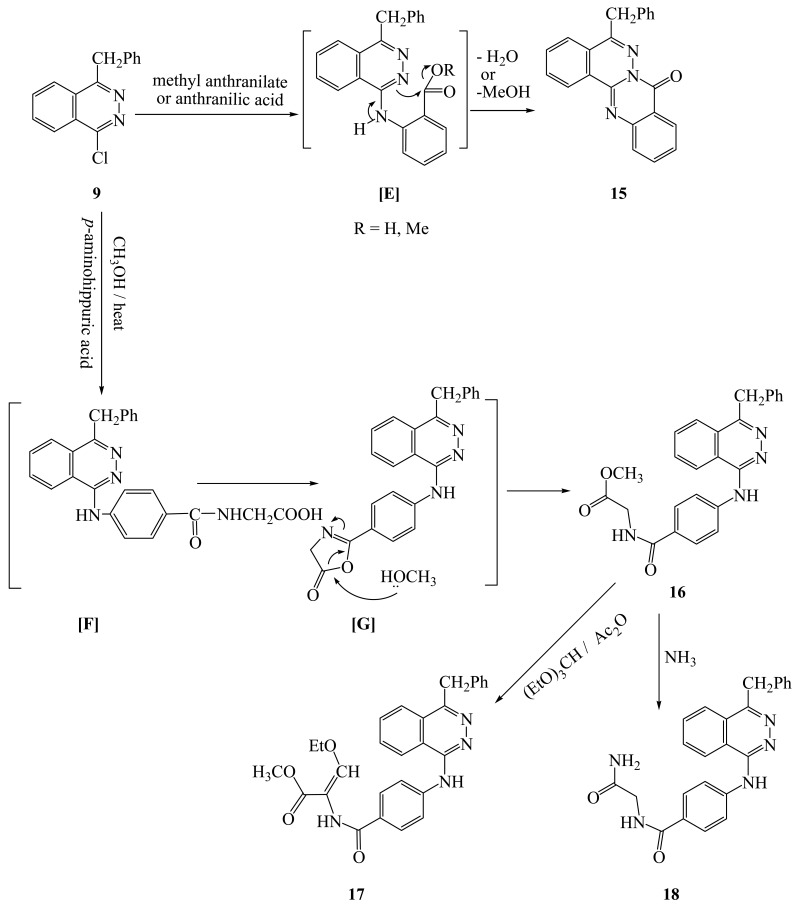
Synthesis of triazobenzobenzo[a]anthrcen-7-one, methyl ester and carboxamide derivatives.

4-Benzyl-1-chlorophthalazine (**9**) [19] was used as starting martial from preparation of new 1,4-disubstuted phthalazine derivatives. Thus, interaction of **9** with equimolar amount of aminophenols, aminoacetophenones and *p*-aminobenzoic acid gave the corresponding 4-benzylphthalazin-1-ylamino derivatives (**10a-f**). Compound **9** also reacted with *N*-(4-aminophenyl)acetanilide under reflux to give the corresponding 4-(4-benzylphthalazin-1-ylamino)acetanilide (**10g**), in the case of *p*-phenylene-diamine and/or benzidine but in the case of *p*-aminodiphenyl amine the only isolable product is 4-(4-benzylphthalazin-1-ylamino)diphenylamine (**10h**). Treatment of **9** with sulfanilic acid and/or J-acid (2-amino-5-naphthol-7-sulfonic acid) gave the corresponding 4-(4-benzylphthala-zin-1-ylamino)benzenesulfonic acid (**10i**) and/or 7-(4-benzylphthalazin-1-ylamino)-4-hydroxynaphthalene-2-sulfonic acid (**10j**), while **9** with ammonium thiocyanate gave the corresponding 4-benzylphthalazin-1-ylthiocyanate **(11)**. Interaction of **9** with dibasic aromatic amines in a 1:2 ratio under fusion conditions afforded the corresponding *N,N′*-bis(4-benzyl-phthalazin-1-yl)-1,4-phenylenediamine and/or *N,N′*-bis(4-benzylphthalazin-1-yl)biphenyl-4,4′-diamines (**12a**,**b**). Also, **9** was reacted with ethylenediamine in boiling ethanol to give *N,N′*-bis(4-benzylphthalazin-1-yl)ethane-1,2-diamine (**13**), However, interaction of **9** with 2-hydrazino-4-amino-5-cyano-6-arylpyrimidine gave the corresponding pyrimidine-5-carbonitrile derivatives **14a,b** respectively (Scheme 2). Interaction of **9** with methyl anthranilate and/or anthranilic acid gave anthracen-7-one derivative **15** as the only isolable product. The formation of **15** was explained by the formation of intermediate [**E**] which undergoes intramolecular ring closure to the final product **15** (Scheme 3).

Treatment of **9** with *p*-aminohippuric acid in boiling methanol/TEA, gave the corresponding methyl ester derivative **16** was obtained as the only isolable product. The formation of **16** can be explained by the nucleophilic transformation into benzylphthalazin-1-ylamino derivative [**F**] as intermediate which undergoes intramolecular cyclization into oxazolone intermediate [**G**] then reacted with a molecule of methanol to give the methyl ester derivative **16** as the final product (Scheme 3).

Treatment of **16** with (EtO)_3_CH/Ac_2_O under reflux afforded the corresponding ethoxymethylene derivative **17**. Treatment of **16** with aqueous ammonium hydroxide under reflux in dioxane in the presence of triethylamine afforded the corresponding carboxamide derivative **18** respectively (Scheme 3).

## 3. Experimental

### 3.1. General

Melting points were determined on a Stuart melting point apparatus and are uncorrected. IR spectra were recorded in KBr using a FT-IR 5300 spectrometer and Perkin Elmer spectrum RXIFT-IR system (ν, cm^−1^). The ^1^H NMR at (300 MHz) and ^13^C NMR spectra (75 MHz) were recorded in CDCl_3_ or DMSO-*d_6_* on a Varian Mercury VX-300 NMR spectrometer. Chemical shifts (δ) are related to that of the solvent. Mass spectra were measured on a Shimadzu GMMS-QP-1000 EX mass spectrometer at 70 eV. The elemental analyses were performed at the Microanalytical Center, Cairo University. Cairo (Egypt).

*4-Benzyl-2-hydroxymethyl-2H-phthalazin-1-one* (**2**). A mixture of compound **1** (0.23 g, 1.0 mmol) and formaldehyde solution 38% (0.8 mL) in ethanol (30 mL) was refluxed for 3 hours. The solvent was evaporated under vacuum, and then water (25 mL) was added. The solid obtained was filtered off and recrystallized from ethanol. Colourless crystals, 90%, 0.19 g. mp 115-116 °C; IR (ν_max_, cm^−1^): 3334 (OH), 1640 (CO). ^1^H NMR (CDCl_3_): δ_H_ 4.13 (t, *J* = 8.4 Hz, 1H, OH), 4.31 (s, 2H, CH_2_Ph), 5.69 (d, *J* = 8.4 Hz, 2H, CH_2_OH), 7.30 (m, 5H, Ph-H), 7.71 (m, 3H, phthalazinyl-3H), 8.45 (m, 1H, phthalazinyl-1H); the singlet at 4.134 was cancelled by D_2_O. ^13^C NMR (DMSO-d_6_) δ 37.3 (CH_2_), 60.1 (CH_2_OH), 125.7 (C_4_-Ar), 128 (C_5_), 128.3 (C_8_), 128.8 (C_3_-Ar, C_5_-Ar), 129.1 (C_2_-Ar, C_6_-Ar), 130.2 (C_5a_), 130.3 (C_8a_), 131.2 (C_7_), 132.3 (C_6_), 137.2 (C_1_-Ar), 155.1 (C_4_) and 160.1 (CO). MS, *m/z* (%) = 266 (M^+^, 10.25), 235 (100) together with other peaks at 207 (5.74), 178 (20.38), 149 (15.82), 130 (9.19), 91 (12.03), 57 (17.77); Anal. Calcd for C_16_H_14_N_2_O_2_ (266.10): C, 72.16; H, 5.30; N, 10.52%. Found: C, 71.89; H, 5.02; N, 9.96%.

*4-Benzyl-2-chloromethyl-2H-phthalazin-1-one* (**3**). A mixture of compound **2** (0.26 g, 1.0 mmol) and thionyl chloride (5 mL) was refluxed for 1 hour. The solid obtained was filtered off and recrystallized from benzene. Colourless crystals, 87%, 0.12 g. mp 125-127 °C; IR (ν_max_, cm^−1^): 3046 (C-H aromatic), 1662 (CO). MS, *m/z* (%) = 284 (M^+^, 43.4), M+2 (12.7), 249 (100) together with other peaks at 220 (34.9), 178 (17.9), 130 (29.2), 91 (80.7), 51 (28.9); Anal. Calcd for C1_6_H_13_ClN_2_O (284.07): C, 67.49; H, 4.60; N, 9.84%. Found: C, 66.90; H, 4.08; N, 9.11%.

*4-Benzyl-2-thiocyanatomethyl-2H-phthalazin-1-one* (**4**). A mixture of compound **3** (0.28 g, 1.0 mmol) and potassium thiocyanate (1.0 mmol) in ethanol (30 mL) was refluxed for 3 hours. The solvent was evaporated under vacuum. The solid obtained was filtered off and recrystallized from ethanol. White crystals, 85%, 0.17 g. mp 155-156 °C; IR (ν_max_, cm^−1^): 2158 (SCN), 1664 (CO). MS, *m/z* (%) = 307 (M^+^, 0.7), 249 (56.3), 130 (12.8) and 91 (100); Anal. Calcd for C_17_H_13_N_3_OS (307.36): C, 66.43; H, 4.26; N, 13.67%. Found: C, 66.22; H, 3.98; N, 13.50%.

*4-Benzyl-2-mercaptomethyl-2H-phthalazin-1-one* (**5**). A mixture of compound **3** (0.28 g, 1.0 mmol) and thiourea (1.0 mmol) in ethanol (30 mL) was refluxed for 3 h hours. The solvent was evaporated under vacuum. The solid obtained was filtered off and recrystallized from ethanol. White crystals, 80%, 0.10 g. mp 145-146 °C; IR (ν_max_, cm^−1^): 2750 (SH), 1650 (CO). MS, *m/z* (%) = 282 (M^+^, 17.2), 249 (78.2), 235 (18.1), 132 (27.3), 130 (23.5), 91 (100) 51 (32.8); Anal. Calcd for C_16_H_14_N_2_OS (282.36): C, 68.06; H, 5.00; N, 9.92%. Found: C, 67.45; H, 4.67; N, 9.22%.

*Ethyl (4-benzyl-1-oxo-1H-phthalazin-2-yl)acetate* (**6**). A mixture of compound **1** (0.23 g, 10 mmol) and ethyl chloroacetate (2 mL) and anhydrous K_2_CO_3_ (0.13 g, 1.0 mmol) was refluxed for 4 hours. The solvent was evaporated under vacuum, then water (50 mL) was added. The solid obtained was filtered off and recrystallized from pet. ether and chloroform, respectively. Violet crystals, 65%, 0.13 g. mp 76-78 °C; IR (ν_max_, cm^−1^): 2978 (CH aliphatic), 1744, 1648 (CO). MS, *m/z* (%) = 322 (M^+^, 37.9) and base peak at 248 together with other peak at 249 (97.9), 221 (8.2), 220 (21.5), 219 (24.3), 193 (4.6), 102 (14.6), 91 (98.4), 76 (11.4); Anal. Calcd for C_19_H_18_N_2_O_3_ (322.12): C, 70.79; H, 5.63; N, 8.69%. Found: C, 70.36; H, 5.44; N, 8.52%.

*(4-Benzyl-1-oxo-1H-phthalazin-2-yl)acetic acid hydrazide* (**7**). A mixture of compound **6** (0.32 g, 10 mmol) and hydrazine hydrate (0.8 mL) in ethanol (30 mL) was refluxed for 3 hours. The solvent was evaporated under vacuum, then water (25 mL) was added. The solid obtained was filtered off and recrystallized from ethanol. White crystals, 86%, 0.18 g. mp 130-132 °C; IR (ν_max_, cm^−1^): 3326 (NH), 1684 (CO, carboxylic acid hydrazide), 1642 (CO, phthalazinyl). ^1^H NMR (CDCl_3_): δ_H_ 14.29 (s, 2H, CH_2_Ph), 4.75 (s, 2H, NCH_2_CO), 7.29 (m, 5H, Ar-H), 7.81 (m, 5H, phthalazinyl-3H and NH_2_), 8.25 (m, 1H, phthalazinyl-H) and 9.30 (s, 1H, CONH; cancelled by D_2_O). MS, *m/z* (%) = 308 (M^+^, 0), 278 (7.2), 277 (23.4), 249 (44.9), 130 (15.3), 91 (100); Anal. Calcd for C_17_H_16_N_4_O (308.33): C, 66.22; H, 5.23; N, 18.17%. Found: C, 66.09; H, 5.12; N, 17.89%.

### 3.2. General Procedure for the Synthesis of Benzylphthalazin-1-ylamino Derivatives **10a-h**

A mixture of compound **9** (0.25g, 10 mmol) and aromatic amine (10 mmol) in ethanol (30 mL) was refluxed for 3 hours. The solvent was evaporated under vacuum. The solid obtained was filtered off to give crude products

*(4-Benzylphthalazin-1-ylamino)phenols (10a,b)*. Compound **10a** Yellow crystals, 90%, 0.17 g. mp 240-242 °C; IR (ν_max_, cm^−1^): 3258 (NH and OH phenolic) as broad peak. MS, *m/z* (%) = 307 327 (M^+^, 42.2) and the base peak at 326 together with other peaks at 325 (84.3), 91 (62.6); Anal. Calcd for C_21_H_17_N_3_O (327.10): C, 77.04; H, 5.23; N, 12.84%. Found: C, 76.83; H, 5.02; N, 12.56%. Compound **10b**: Yellow crystals, 80%, 0.12 g. mp 236-237 °C; IR (ν_max_, cm^−1^): 3268 (NH and OH phenolic) as broad peak. MS, *m/z* (%) = 327 (M^+^, 40.0) and the base peak at 326 together with other peaks at 325 (86.7), 91 (50.8); Anal. Calcd for C_21_H_17_N_3_O (327.10): C, 77.04; H, 5.23; N, 12.84%. Found: C, 76.75; H, 5.01; N, 12.59%.

*7-(4-Benzylphthalazin-1-ylamino)naphthalen-2-ol* (**10c**). Green crystals, 70%, 0.09 g. mp 242-243 °C; IR (ν_max_, cm^−1^): 3272 (OH and NH) as broad peak. ^1^H NMR (DMSO-*d_6_*): δ_H_ 24.65 (s, 2H, CH_2_Ph), 7.08-7.33, 7.55-7.58, 8.09-8.11 (ms, 9H, Ar-H), 7.38 (d, *J* = 7.8 Hz, 1H, Ar-H), 7.78 (d, *J* = 9 Hz, 1H, Ar-H), 7.86 (d, *J* = 9 Hz, 1H, Ar-H), 8.14 (d, *J* = 1.8 Hz, 1H, Ar-H), 8.16 (s, 1H, NH, exchangeable with D_2_O), 8.33 (d, *J* = 1.8 Hz, 1H, Ar-H), 9.04 (d, *J* = 7.8 Hz, 1H, Ar-H) and 9.96 (s, 1H, phenolic-OH, exchangeable with D_2_O).; Anal. Calcd for C_25_H_19_N_3_O (377.13): C, 79.55; H, 5.07; N, 11.13%. Found: C, 79.32; H, 4.87; N, 10.92%.

*1-[(4-Benzylphth-alazin-1-ylamino)phenyl]ethanones* (**10d,e**). Compound **10d**: White crystals, 75%, 0.11 g. mp 225-226 °C; IR (ν_max_, cm^−1^): 3390 (NH), 1662 (CO). MS, *m/z* (%) = 353 (73.56) and the base peak at 352 together with other peaks at 311 (3.7), 310 (11.97), 262 (10.56), 220 (5.37), 219 (4.82) and 91 (54.14); Anal. Calcd for C_23_H_19_N_3_O (353.05): C, 78.16; H, 5.42; N, 11.89%. Found: C, 77.88; H, 5.23; N, 11.64%. Compound **10e**: Yellow crystals, 80%, 0.13 g. mp 260-261 °C; IR (ν_max_, cm^−1^): 3312 (NH), 1670 (CO). ^1^H NMR (DMSO-*d_6_*): δ_H_ 2.57 (s, 3H, CH_3_CO), 4.75 (s, 2H, CH_2_Ph), 7.21-7.27 (m, 2H, Ar-H), 7.31 and 7.40 (AB-q, *J* = 7.2 Hz, 4H, Ar-H), 8.01 (s, 1H, NH), 8.10-8.18 (m, 5H, Ar-H) and 8.39, 8.94 (2d, *J* = 7.8 Hz, 2H, Ar-H); Anal. Calcd for C_23_H_19_N_3_O (353.23): C, 78.18; H, 5.42; N, 11.89%. Found: C, 77.97; H, 5.18; N, 11.69%.

*4-(4-Benzylphthalazin-1-ylamino)benzoic acid* (**10f**). Yellow crystals, 85%, 0.16 g. mp 280-281 °C; IR (ν_max_, cm^−1^): 3224 (NH and OH), 1676 (CO). MS, *m/z* (%) = 355 (38.4) and base peak at 354 together with other peaks at 310 (3), 234 (5.3), 204 (9.9), 219 (6.9), 204 (9.9), 178 (10.1), 102 (15.9), 91 (68.3), 90 (60.2), 77 (30.6) and 76 (29.7); Anal. Calcd for C_22_H_17_N_3_O_2_ (355.16): C, 74.35; H, 4.82; N, 11.82%. Found: C, 74.14; H, 4.57; N, 11.60%.

*4-(4-Benzylphthalazin-1-ylamino)diphenyl amine* (**10g**). Green crystals, 70%, 0.13 g. mp 215-217 °C; IR (ν_max_, cm^−1^): 3276 (NH). MS, *m/z* (%) = 402 (M^+^, 46.5), 117 (88.7), 92 (60.32), 76 (28.37), 91 (18.88), 57 (87.49), 56 (29.88) & 55 (100); Anal. Calcd for C_27_H_22_N_4_ (402.02): C, 80.57; H, 5.51; N, 13.92%. Found: C, 80.44; H, 5.49; N, 13.75%.

*4-(4-benzylphthalazin-1-ylamino)acetanilide* (**10h**). Yellow crystals, 80%, 0.18 g. mp > 340 °C; IR (ν_max_, cm^−1^): 3428 (NH), 1620 (CO). MS, *m/z* (%) = 368 (M^+^, 12.94), 235 (10.25), 149 (88.35), 117 (78.7), 92 (63.32), 76 (28.37), 91 (18.88), 57 (87.49), 56 (29.88) and 55 (100); Anal. Calcd for C_23_H_20_N_4_O (368.17): C, 74.98; H, 5.47; N, 15.21%. Found: C, 74.80; H, 5.32; N, 15.12%.

### 3.3. General Method for the Synthesis of Sulfonic Acid Derivatives **10i, j**

A mixture of compound **9** (0.25 g, 1.0 mmol) in ethanol (30 mL) sulfanilic acid or (2-amino-5-naphthol-7-sulfonic acid) (1.0 mmol) and 2 drops of triethylamine (TEA) was refluxed for 3 hours. The solvent was evaporated under vacuum. The solid obtained was filtered off and recrystallized from dioxane.

*4-(4-Benzylphthalazin-1-ylamno)benzenesulfonic acid* (**10i**). Yellow crystals, 80%, 0.17 g. mp > 340 °C; IR (ν_max_, cm^−1^): 3402 (OH), 3256 (NH). MS, *m/z* (%) = 391 (M^+^, 4.7), 327 (18.12), 311 (72.15), 310 (95.64), 235 (40.60), 219 (89.60), 193 (100), 92 (57.72) and 91 (16.78). Anal. Calcd for C_21_H_17_N_3_O_3_S (391.09): C, 64.43; H, 4.38; N, 10.73%. Found: C, 64.36; H, 4.28; N, 10.65%.

*7-(4-benzylphthalazin-1-ylamino)-4-hydroxynaphthalene-2-sulfonic acid* (**10j**). Yellow crystals, 65%, 0.13 g. mp > 340 °C; IR (ν_max_, cm^−1^): 3308 (OH), 3066 (NH). MS, *m/z* (%) = 457 (M^+^, 5.3), 455 (naphthsaltone cationic radical, 59.1), 222 (5.1), 235 (100), 203 (6.4), 193 (3.6) and 178 (15.8); Anal. Calcd for C_25_H_19_N_3_O_4_S (457): C, 65.63; H, 4.19; N, 9.18%. Found: C, 65.54; H, 3.97; N, 9.10%.

*Synthesis of N,N′-bis(4-benzylphthalazin-1-yl)-1,4-phenylenediamine* (**11**) *and N,N′-bis(4-benzylphthalazin-1-yl)biphenyl-4,4′-diamine* (**12**). A mixture of compound **9** (0.50 g, 20 mmol) and dibasic aromatic amines (10 mmol) in ethanol (30 mL) was refluxed for 3 hours. The solvent was evaporated under vacuum. The solid obtained was filtered off and recrystallized from dioxane. Compound **11** Yellow crystals, 70%, 0.13 g. mp 250-251 °C; IR (ν_max_, cm^−1^): 3344 (NH). MS, *m/z* (%) = 544 (M^+^, 0.74) and base peak at 325 together with other peaks at 234 (5.7), 219 (3.29), 106 (1.74), 91 (13.66), 76 (1.25); Anal. Calcd for C_36_H_28_N_6_ (544.27): C, 79.39; H, 5.18; N, 15.43%. Found: C, 79.08; H, 5.06; N, 15.22%. Compound **12**: Yellow crystals, 70%, 0.12 g. mp > 340 °C; IR (ν_max_, cm^−1^): 3277 (NH). MS, *m/z* (%) = 620 (M^+^, O), M/2 (310, 9.3); the base peak at 401 and other peaks at 102 (1.8), 91 (17.1); Anal. Calcd for C_42_H_32_N_6_ (620.13): C, 81.27; H, 5.20; N, 13.54%. Found: C, 80.96; H, 5.04; N, 13.30%.

*N,N′-bis(4-benylphthalazin-1-yl)ethane-1,2-diamine* (**13**). A mixture of compound **9** (0.50 g, 20 mmol) and ethylenediamine (10 mmol) in absolute ethanol (30 mL) anhydrous K_2_CO_3_ was refluxed for 4 hours. The obtained was cooled to room temperature, then diluted with water. The solid obtained was filtered off and recrystallized from ethanol. White crystals, 60%, 0.21 g. mp 234-235 °C; IR (ν_max_, cm^−1^): 3316, 3260 (NH) 2930, 2870 (C-H aliphatic). ^1^H NMR (DMSO-*d_6_*): δ_H_ 3.93 (s, 4H, CH_2_-CH_2_), 4.48 (s, 4H, 2CH_2_Ph), 7.14-7.32 (m, 10H, Ar-H), 7.78-7.28 (m, 4H, Ar-H), 7.93-7.90 (br s, 2H, NH; exchangeable with D_2_O), 8.00-8.021(m, 2H, Ar-H) and 8.26-8.29 (m, 2H, Ar-H); Anal. Calcd for C_32_H_28_N_6_ (496.60): C, 77.39; H, 5.68; N, 16.92%. Found: C, 76.29; H, 5.45; N, 16.65%.

### 3.4. General Method for the Synthesis of Hydrazino Pyrimidine Derivatives **14a,b**

A mixture of compound **9** (0.25 g, 10 mmol) and 2-hydrazino-4-amino-5-cyano-6-arylpyrimidine (10 mmol) in *n*-butanol (30 mL) was refluxed for 3 hours. The solvent was evaporated under vacuum. The solid obtained was filtered off and recrystallized from dioxane.

*4-Amino-2-[N′-(4-benzylphthalazin-1-yl)hydrazino]-6-(4-methoxyphenyl)-pyrimidine-5-carbonitrile* (**14a**). White crystals, 75%, 0.16 g. mp 294-295 °C; IR (ν_max_, cm^−1^): 3450, 3210 (NH), 2206 (CN), 1634 (C=N). ^1^H NMR (DMSO-*d_6_*): δ_H_ 3.84 (s, 3H, OCH_3_), 4.58 (s, 2H, CH_2_Ph), 7.10 and 7.13 (2d, *J* = 8.4 Hz), 2H, Ar-H), 7.17 and 7.22 (2d, *J* = 7.8 Hz, 2H, Ar-H), 7.37 and 7.57 (2d, *J* = 7.8 Hz, 4H, Ar-H), 8.18 and 8.46 (2d, *J* = 8.1 Hz, 2H, Ar-H), 8.684 (s, 1H, Ar-H), 6.99 and 7.88 (m, 2H, Ar-H), 6.250 (br s, 2H, NH_2_), 8.90 (br s, 2H, 2-NH); Anal. Calcd for C_27_H_22_N_8_ (474.02): C, 68.34; H, 4.67; N, 23.612%. Found: C, 68.34; H, 4.55; N, 23.54%.

*4-Amino-2-[N′-(4-benzylphthalazin-1-yl)hydrazino]-6-(4-chorophenyl)pyramidine-5-carbonitrile* (**14b**). White crystals, 80%, 0.19 g. mp 274-275 °C; IR (ν_max_, cm^−1^): 3380, 3270 (NH), 2192 (CN), 1638 (C=N). MS, *m/z* (%) = 478 (M^+^, and M+2), 462 (19.22), 368 (14.61), 236 (12.68), 275 (75.31), 138 (36.35), 128 (46.12), 111 (29.49), 91(100); Anal. Calcd for C_26_H_19_ClN_8_ (478.01): C, 65.20; H, 4.00; N, 23.40%. Found: C, 65.08; H, 3.87; N, 23.29%.

*5-Benzyl-6,6a,12-triazobenzo[a]anthracen-7-one* (**15**). A mixture of compound **9** (0.25 g, 10 mmol) and methyl anthranilate or anthranilic acid (10 mmol) in absolute ethanol (30 mL) and 2 drops of triethylamine (TEA) was refluxed for 3 hours. The solvent was evaporated under vacuum. The solid obtained was filtered off and recrystallized from dioxane. Yellow crystals, 80%, 0.16 g. mp 176-178 °C; IR (ν_max_, cm^−1^): 1704 (CO); MS, *m/z* (%) = 337 (M^+^, 66.92), 336 (100) and 91 (13.09); Anal. Calcd for C_22_H_15_N_3_O (337.12): C, 78.32; H, 4.48; N, 12.46%. Found: C, 78.19; H, 4.37; N, 12.30%.

*[4-(4-Benzylphthalazin-1-ylamino)benzoylamino]acetic acid methyl ester* (**16**). A mixture of compound **9** (0.25 g, 10 mmol) and *p*-aminohippuric acid (10 mmol) in methanol (30 mL) and three drops of TEA was refluxed for 3 hours. The solvent was evaporated under vacuum. The solid obtained was filtered off and recrystallized from dioxane. Yellow crystals, 90%, 0.18 g. mp 165-167 °C; IR (ν_max_, cm^−1^): 3110, 3328 (NH), 1752 (CO), 1642 (CONH). ^1^H NMR (DMSO-*d_6_*): δ_H_ 3.67 (s, 3H, OCH_3_), 3.95 (d, *J* = 5.4 Hz), 1H, non equivalent CH_2_ protons), 4.04 (d, *J* = 5.4 Hz), 1H, non equivalent CH_2_ protons), 4.73 (s, 2H, PhCH_2_), 7.88 and 8.006 (2d, *J* = 8.7 Hz, 2H, Ar-H), 8.44 (d, *J* = 7.8 Hz, 1H, Ar-H), three sets of multipletes at (7.24-7.30), (8.14-8.28), (8.93-9.01) (6H, Ar-H and NH), 7.33 and 7.41 (AB-q, *J* = 7.2Hz, 4H, Ar-H), 10.80 (hump, 1H, NH, exchangeable by D_2_O).; MS, *m/z* (%) = 426 (M^+^, 48.2), 425 (100), 338 (27.8), 310 (5.4), 219 (2.7), 128 (5.4) and 91 (37.2).; Anal. Calcd for C_15_H_22_N_4_O_3_ (426.46): C, 70.41; H, 5.20; N, 13.14%. Found: C, 70.25; H, 5.07; N, 12.87%.

*Methyl 2-(4-(4-benzylphthalazin-1-ylamino)benzamido)-3-ethoxyacrolate* (**17**). A mixture of compound **16** (0.42 g, 1.0 mmol) and triethyl orthoformate (0.14 g, 1.0 mmol) in acetic anhydride (10 mL) was refluxed for 5 hours, and then allowed to cool at room temperature and diluted with water (20 mL). The solid obtained was filtered off and recrystallized from dioxane Yellow crystals, 60%, 0.17 g. mp 175-177 °C; IR (ν_max_, cm^−1^): 3060 (C-H olefinic) 3366 (NH), 2942 (C-H aliphatic), 1752, 1666 (CO). ^1^H NMR (DMSO-*d_6_*): δ_H_ 2.08 (t, 3H, CH_2_CH_3_), 3.64 (s, 3H, COOCH_3_), 4.01 (q, 2H, CH_2_CH_3_), 4.75 (s, 2H, CH_2_Ph), (7.17-8.38) (sets of multiplets, 15H, Ar-H and 2NH), 8.97 (s, 1H, =CH); Anal. Calcd for C_28_H_26_N_4_O_4_ (482.52): C, 69.70; H, 5.43 N, 11.49%. Found: C, 69.63; H, 5.23; N, 11.49%.

*2-(4-(4-Benzylphthalazin-1-ylamino)benzamido)acetamide* (**18**). To compound **16** (0.42 g, 1.0 mmol) and distilled water and/or ammonium hydroxide, two drops of triethylamine (TEA) were added, and the mixture was refluxed in dioxane (15 mL) for 2 hours. The solvent was evaporated under vacuum. The solid obtained was filtered off and recrystallized from dioxane. Yellow crystals, 75%, 0.19 g. mp 240-242 °C; IR (ν_max_, cm^−1^): 3272, 3354(NH), 1734, 1640 (CO); MS, *m/z* (%) = 411 (55.9), 394 (20.7), 355 (59), 354 (100), 338 (48.0), 246 (32.8); Anal. Calcd for C_24_H_21_N_5_O_2_ (411): C, 70.06; H, 5.14; N, 17.02%. Found: C, 69.89; H, 5.07; N, 16.93%.

### 3.5. Antibacterial Activity

The newly synthesized compounds were screened for their antimicrobial activities *in vitro* against two species of Gram-negative bacteria *Pseudomonas aeruginosa* (MTCC 741); *Escherichia coli* (NCTC-10410); and four Gram-positive bacteria, *Bacillus cereus* (ATGG 14579); *Bacillus subtilis* (MTCC 441); *Bacillus sphaericus* (MTCC 11); *Staphylococcus* (MTCC 96); and two fungus, *Aspergillus ochraceus Wilhelm* (AUCC-230); *Penicillium chrysogenum Thom* (AUCC-530). The activities of these compounds were tested using the disc diffusion method [20]. For bacteria and the paper disk diffusion method [21] for fungi. The area of zone of inhibition was measured using Ampicillin; tetracycline and norfloxacin (30 μg mL^−1^) as standard antibiotic and mycostatin (30 μg mL^−1^) was used as a reference antifungal. The tested compounds were dissolved in *N,N*-dimethylformamide (DMF) to give a solution of 1 mg mL^−1^. The inhibition zones were measured in millimeters at the end of an incubation period of 48 hours at 28 °C. *N,N*-dimethylformamide (DMF) showed no inhibition zone. Test results are shown in Table 1

### 3.6. Antibacterial Activities

The screening results indicate that compounds **2–8** and **11** show weaker inhibitory activity than the standard drugs, while compounds **15–18** are moderately inhibitory, compared to the standard drugs. Compounds **10a–j**, **12**, **13 and 14a,b** showed nearly the same inhibition activity asn antibacterial activity (Table 1). It clear that decomposition of chloride atom at C-1 of 4-benzyl-1-chlorophthalazine with N-nucleophiles is responsible for the antimicrobial activities.

## 4. Conclusions

The results from this screening demonstrated that replacement of the hydrogen atom attached to the phthalazine nucleus at N-1 with amino derivatives (compounds **10a-j**), diimino derivatives (compounds **12a,b-13**) and pyrimidine derivatives (**14a,b**) resulted in spectrum if moderate antibacterial activity against all tested Gram positive and Gram negative fungi.

## Figures and Tables

**Table 1:** Antimicrobial activity of the new compounds.

**Compd. No. a**	**Inhibition zone diameter in mm**							
	**Gram-negative bacteria**		**Gram-positive bacteria**				**Fungi**	
	**PA**	***EC***	**BC**	**BS**	**BS**	**SC**	**AOW**	**PCT**
**1**	10	12	10	14	12	10	11	13
**2**	14	10	12	11	10	13	10	11
**3**	10	10	14	12	11	10	14	10
**4**	13	10	12	13	10	12	10	12
**5**	11	14	10	13	14	13	12	13
**6**	14	10	13	10	12	14	11	14
**7**	10	12	15	12	10	11	10	11
**8**	12	13	10	14	10	14	10	10
**10a**	16	15	14	15	15	16	17	15
**10b**	18	19	18	17	15	19	14	17
**10c**	16	16	19	15	18	14	17	18
**10d**	15	17	16	19	17	15	16	14
**10e**	17	16	14	17	19	16	19	19
**10f**	16	15	17	18	17	14	15	17
**10g**	18	15	15	16	16	17	18	16
**10h**	19	18	14	15	17	15	14	19
**10i**	17	16	17	19	18	15	17	15
**10j**	18	15	19	16	18	15	16	14
**11**	12	10	14	12	10	14	10	11
**12a**	20	22	20	19	20	22	20	20
**12b**	20	23	22	24	24	20	20	20
**13**	22	20	23	22	23	20	24	22
**14a**	23	22	24	23	22	24	23	23
**14b**	24	20	20	20	22	24	20	22
**15**	19	22	23	22	24	20	20	21
**16**	18	17	18	17	19	18	16	17
**17**	17	19	16	19	17	19	15	16
**18**	15	18	15	17	18	16	18	15
**Ampicillin**	22	22	22	22	22	22	-	-
**Tetracycline**	20	20	20	20	20	20	-	-
**Norfloxacin**	25	25	25	25	25	25	-	-
**Mycostatin**	-	-	-	-	-	-	20	20

a *c* = 1 mg mL^−1^ of new compounds in DMF. Microorganisms: *Pseudomonas aeruginosa* (MTCC 741); *Escherichia coli* (NCTC-10410); *Bacillus cereus* (ATGG 14579); *Bacillus subtilis* (MTCC 441); *Bacillus sphaericus* (MTCC 11); *Staphylococcus* (MTCC 96); *Aspergillus ochraceus Wilhelm* (AUCC-230); *Penicillium chrysogenum Thom* (AUCC-530).
